# Ballistic Trauma of Limbs

**DOI:** 10.2174/1874325001711010268

**Published:** 2017-03-31

**Authors:** Léopold Lamah, Damany Keita, Ibrahima Marie Camara, Mohamed Lamine Bah, Sidimé Sory, Mamadou Moustapha Diallo

**Affiliations:** University of Conakry Gamal Abdel Nasser - Medecine Hospital Donka, Conakry 00224, Guinea

**Keywords:** Ballistics, Case special, Donka members, Traumatism, Traumatology

## Abstract

The objective of our study was to report the management and follow-up of a particular case of ballistic trauma and to do the literature review.

**Observation:**

A 35-year-old patient, a trader who was the victim of a firearm accident under not very clear circumstances. He was admitted to the emergency department after 3 hours. Clinically, the patient had significant bleeding in the arm and was in a state of clouding of consciousness. We could notice on the right arm, a posterior large transfixing wound of 1 cm and a 6 cm one on the antero-internal side. The limb was cold with a small and thready pulse. Sensitivity was decreased in the radial nerve area. The radiograph showed bone comminution from the middle 1/3 to the superior 1/3 of the humeral diaphysis. The treatment was orthopedic (after debridement) by scapula-brachio-ante-brachiopalmar plaster splint with thoracic strap. The wound healed in 46 days and the patient resumed his activities after 11 months and 2 weeks.

**Conclusion:**

The authors presented the value of using the scapulo-brachio-palmar plaster splints with thoracic strap in some severe upper limb trauma in the absence of the external fixator.

## INTRODUCTION

The limbs are the most frequent anatomical location of ballistic trauma [1]. These injuries result in lesions ranging from simple wounds to severe trauma or even traumatic amputations. The victims are young people, with an average age of 21.7 years, who have been attacked [2]. Increasingly frequent, these injuries pose management problem in the developing countries where resources are limited.

Any thesis makes it possible to predict with certainty the behavior of a projectile in the human organism [3]. Although there is now consensus on the modalities of preoperative resuscitation of hemorrhagic injured patients, the surgical strategy is not unequivocal [1]. This surgical strategy poses many difficulties and varies from one medical department to another, from one country to the other depending on the means available.

The objective of our study was to expose the case of a ballistic trauma in which bone lesion was not ordinary and to show our therapeutic strategy.

### Presentation of Case

Mr. O D, 35 years old, trader, admitted to the emergency department on April 22nd, 2013.

Chief complaints: open right arm trauma

Evolution: 3 hours

Circumstances: He was allegedly wounded by firearm under not very clear circumstances at 3 AM.

Overall checkup: The patient was admitted in a state of unconsciousness and cold sweat. The teguments and conjunctivae were pale.The thorax was symmetrical and breathing was polypneic with 32 beats per minute.The pulse was small and thready, and the heart sounds were muffled. The heart rate was of 112 / minute. In shock, the patient blood pressure was 8/5 mmhg. The abdomen was supple and participating in breathing, with no palpable pathological mass.

Elsewhere, the other organs showed no special characteristics.

### Loco-Regional Assessment

The right upper limb was protected by a cloth soaked in blood. The limb was swollen and covered with dust and blood. With the removal of the linen, we noticed a transfixing wound in the arm, with a posterior entry point of 1 cm and an antero-internal exit point of 6 cm. At the junction of the middle third and the superior third, we observed telluric debris and small pieces of fabric in the wound. We also noticed other superficial wounds opposite to the posterior surface of the thorax. The radial pulse was perceived but small. The neurological examination was difficult because of the patient's state of consciousness.

### Emergency Management

We made a quick cleaning with physiological saline serum, made a large sterile dressing on the wound, put two venous lines. On one side, we put the lactate ringer, and on the other side, we put plasma. A 1500 IU anti-tetanus serum ampoule was injected subcutaneously; 2 grams of ampicillin IV and anampoule of gentamycin 80 were given by intramuscular injection.

### Emergency Examinations

**Fig. (1):**
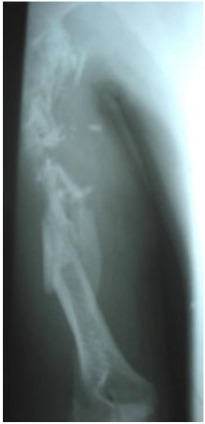
Initial x-ray.

Biological: Blood count (hemoglobin at 8 grams and patient was A +). The postprandial blood glucose level was 1.8 g / dl. X-ray: showed a very comminuted fracture of the humeral diaphysis from the middle third to the superior third (Fig. **1**).

The patient was admitted to the operating room after resuscitation. He was placed in the dorsal recumbent position on an ordinary table and under general anesthesia with a tourniquet at the root of his arm.

While performing the probe, we noticed a loss of 4cm in the diameter of cutaneous substance, an important bone comminution located from the middle third to the superior third which made the fracture unstable with a breach on the humeral artery, a partial section of the radial nerve. We concluded to the diagnosis of type IIIc Gustilo Anderson, open fracture of the middle third and superior third of the humerus bone.

### Operating Technique

We clamped and sutured the breach of the humeral artery and cleaned the limb with saline solution. To clean the wound, we protected it by a compress slide (to prevent losing the small fragments) and instilled 3 liters of saline solution. Blood clots and pieces of fabric were removed. Haemostasis was performed using 2/0 dissolving sutures. Economic resection of the wound edges was carried out, followed by an epiperinural suture on the radial nerve using 4/0 non absorbables sutures. We sutured the biceps fibers to cover the bone fragments. Verification of haemostasis was performed after removal of the tourniquet. We placed a large dressing on the wound without closing it.

**Fig. (2):**
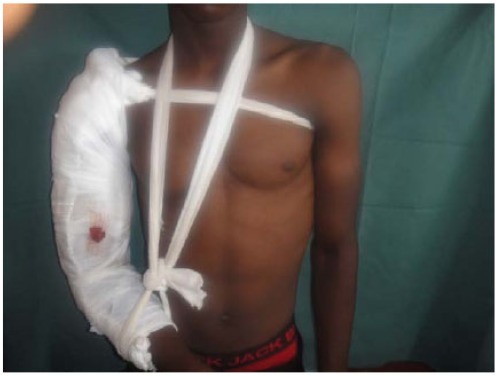
Immobilization of the limb by plastered splint.

The immobilization of the limb was done using scapula-brachio-ante-brachiopalmar plaster splint with thoracic strap (Fig. **2**).

**Fig. (3):**
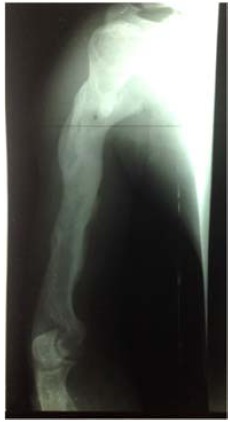
Control x-ray after 48 months.

**Fig. (4):**
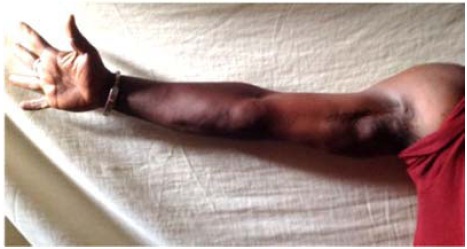
Clinical image showing.

The evolution was marked by superficial infections that we treated by local care and antibiotherapy adapted to the antibiogram. (Staphylococcus aureus, streptococcus and Escherichia coli). The patient was discharged from the hospital after one month and followed by an outpatient basis for 2 months. The patient had complete wound healing on the 46^th^ day. The control X-rays were done on day 3^rd^, 21^st^, 46^th^ and 6^th^ month,

The rehabilitation of the limb began in the 4^th^ month until the 12^th^ month and the patient resumed his activities in the 12^th^ month without sequelae. After 48 months, the patient had no sequelae of his trauma (Figs. **3** and **4**).

## DISCUSSION

### Circumstances

According to Laforge [3], in civil practice, 48% of ballistic injuries are due to assaults, 47% due to suicide and 3% due to accidents. In our patient, the circumstances of the accident were very poorly understood. It was about an aggression or a self-defense on behalf of the perpetrator. We were unable to identify the weapon used by the latter. Among 39 patients treated by Dakouré [4] for ballistic trauma, 58.9% had been accidentally injured.

### Mechanism and Lesions

Displacement of projectiles causes injuries through the transfer of kinetic energy into the human body that destroy, tears and deforms the tissues.

Higher kinetic energy at the entry point implies higher invasive potential. The more this energy is high at the exit point, the lower will be the tissue damages [5]. In our patient, the bone damage was more impressive than the condition of the soft parts. The limitation of the destruction of the soft parts can be explained by the fact that elastic tissues tolerate stretching; however, they badly stand crushing. The destruction of the soft parts is related to the release of kinetic energy. This energy is proportionate to the mass of the projectile. In some cases, the projectile travels a certain distance before releasing its energy [6]. All these phenomena may also explain the limitation of soft tissue damage in our patient. The absence of foreign bodies in the wound testifies that it was about a bullet and not a splinter and the limitation of the crushing of soft parts showed that the bullet had a high energy at its exit point which discarded the soft parts without enough damage [7-9]. The nerves and tendons are movable, the blood vessels are elastic. They were thus moved out of the bullet path while the bullet got into the limb. The cavitation effect depends on the elasticity of the tissues crossed by the bullet. Elastic tissues, such as the lung, easily adapt to temporary deformation. Less elastic structures, such as striated musclesor those contained in rigid envelopes such as the brain, are severely damaged by this wave. The projectile can release a large amount of energy into the bone during its passage. This energy gives a blast effect which spreads along the bone marrow and causes its explosion [10]. This bursting of the bone can propel « secondary projectiles » which also produce tissue damage [11]. The cortical bone, dense and rigid, resists stretching. If the cavitation process accelerates and represses the muscle mass, the bone is curved beyond its strength and breaks [8, 9, 12]. Other experiments have proven that the projectile can go through the tissue without direct contact with the bone but still cause bone fracture.Often it is only observed in simple fractures [10].

### Management and Healing

As with any hemorrhagic wound, the management begins with resuscitation and the urgency of which varies according to the severity of the case. It is mainly about vascular filling. The beneficial effect in terms of the survival of preoperative mass filling by crystalloid or colloidal solutions before obtaining surgical haemostasis is very controversial and some authors recommend initial contributions limited to maintaining a minimum hemodynamic balance before admitting the patient to the operating room [13]. Due to the lack of means, the patient did not undergo arteriogramor doppler echo to specify the gravity of the vascular lesions before debridement. The crucial time of the debridement is the cleaning of the wound, but on a multi-fragmentary bone it is exposed to the risk of losing small fragments. In our patient, we were able to maintain these fragments by protecting the wound by compressing before flushing abundantly with cleaning liquid. If it is true that the periost stripped fragments can become necrotic and turn into sequestrum, it is important to differentiate in our patient in whom, most of the fragments were free. Their extirpation meant to lose some bone matter. These free fragments were rather used as grafts.

This is contrary to other authors, who systematically remove the detached fragments to avoid their sequestration which can maintain the infection [14]. The prerequisite is to wash again and again and then carry out debridement [7] to minimize the infectious risk. Antibiotic therapy can never substitute washing. In the series of Dakouré, all patients undergone debridement and 25.64% were treated orthopedically [4]. The use of external fixators in the treatment of open fractures is th best indication. It also depends on the means and the habits. This method is more resistant,ensures a good stabilityand facilitates local care.In our department, we use the external fixator of the military health unit which does not seem to be well adapted to the upper limb. Hansley [15] used the external fixator in 22% of his patients. Over 54 patients treated for fracture of the humerus by firearm, Vaidya [16] used external fixator for only 6 patients and 25 underwent non operative treatment. The scapulo-brachio-antebrachio-palmar plaster with chest strap that we used has the benefit of immobilizing the shoulder joint which remains stabilized by the chest strap. It does not ensure absolute stability. The micromovements that are allowed by the chest strap in the direction of the axis contribute to speed up the healing process [17]. The healing of the bone depends less on the number of fragments but mainly on the condition of the soft parts. Intraoperative exploration showed limited destruction of the soft parts. The superficial infection that occured during treatment could be due to the sepsis of the bullet, the soiling of the wound (dust and pieces of fabrics) and the conditions of the first aid. The prevention of primary infection is based on an enlarged and early debridement surgery with excision of devitalized and necrotic tissues. The antibiotic therapy is intended to prevent the microbial spread which is rapid and starts from the sixth hour following the trauma. In the series of Hinsley, 13/43 (30.23%) of patients with bullet induced fractures developed infections. Krebsbach [18] demonstrated that the degree of contamination of the wound is inversely proportionate to the speed of the bullet.

## CONCLUSION

Ballistic trauma can be observed in the times of war and in civil practice during the times of peace. In the latter cases, it is mainly about attacks that leave lesions whose nature varies from simple wounds to traumatic amputations. There is still no consensus on the management of these bone lesions. It depends on the lesion, the means available and the emergency.The plaster splints with chest strap are a model of plaster that we find well adapted to the upper limb. In the absence of external fixator, they offer excellent results when they are well used.
